# Supplementary material for: Swimbladder morphology masks Southern Ocean mesopelagic fish biomass
Source: Proc Biol Sci. 2019 May 29;286(1903):20190353. doi: 10.1098/rspb.2019.0353 (PMC6545075; doi:10.1098/rspb.2019.0353)
Supplement: Acoustic backscatter and mesopelagic fish abundance, biomass and swimbladder morphology supplement [file rspb20190353supp1.pdf]

Dornan T, Fielding S, Saunders RA, Genner MJ. 2019 Swimbladder morphology masks Southern Ocean mesopelagic fish biomass. *Proc. R. Soc. B*. (doi: RSPB-2019-0353)

Electronic Supplementary Material

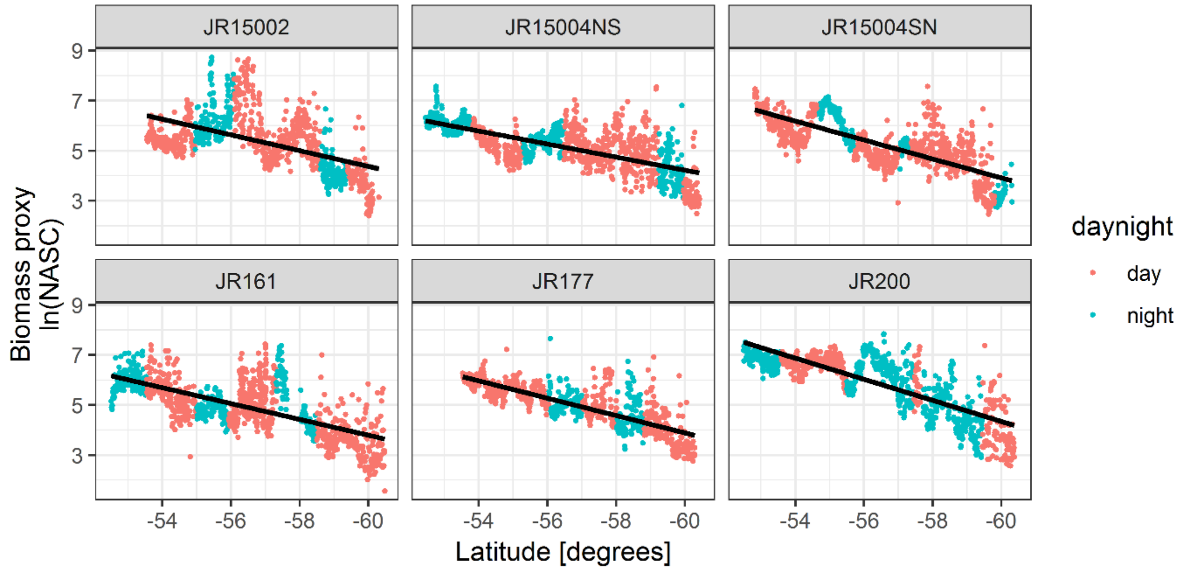

Figure S1. Linear regressions on day night acoustic data. To confirm that diel vertical migration behaviour did not introduce bias, linear regressions on day and night only data were also carried out. Linear regressions continue to reveal highly significant declines in NASC with an increase in latitude ($p < 0.0001$).

Linear regression coefficients of $\log_e$ transformed NASC against latitude.

Data	Cruise	Slope	Std Error	R^2
**All data**	JR15002	0.314709	0.016844	0.279
"	JR15004NS	0.261223	0.008407	0.464
"	JR15004SN	0.378643	0.010354	0.569
"	JR161	0.316616	0.010446	0.453
"	JR177	0.346625	0.009056	0.624
"	JR200	0.421575	0.009866	0.646
**Day only**	JR15002	0.235145	0.019766	0.184
"	JR15004NS	0.211170	0.015136	0.231
"	JR15004SN	0.312942	0.011233	0.497
"	JR161	0.345870	0.013167	0.473
"	JR177	0.357730	0.009004	0.708
"	JR200	0.470532	0.013512	0.760
**Night only**	JR15002	0.556826	0.027631	0.601
"	JR15004NS	0.293638	0.008841	0.703
"	JR15004SN	0.650096	0.013242	0.914
"	JR161	0.223463	0.019327	0.283
"	JR177	0.232736	0.039173	0.135
"	JR200	0.383161	0.013744	0.559

Table S1. Swimbladder gas contents used in analysis. Species: KRA – *Krefftichthys anderssoni*, ELN – *Electrona antarctica*, ELC – *E. carlsbergi*, GYR – Gymnoscopelus braueri, GYF – *G. fraseri*, GYN – *G. nicholsi*, PRM – *Protomyctophum bolini*. Treatment details pre-treatment of fish prior to assessment of swimbladder condition, Frozen: scanned from frozen, IKI: fixed and stained with Potassium Iodide, Fresh: untreated, freshly captured. Gas: Y – Yes, R – Ruptured, N – No, Inc – Inconclusive exclude from analysis, D – Damaged exclude from analysis. Data source is method of assessment, CT – computed tomography, X-ray – Soft tissue x-ray, Dis – dissection of freshly captured fish. Net type indicates sample method of capture, either RMT25 or opportunistic samples from RMT8 or MOCNESS (MOC). Lat and Lon are the mean net sample latitude and longitude during oblique tows respectively.

Cruise	Event	Net	Species	Sex	SL mm	Treatment	Gas	Gas binary	Data source	Net type	Lat	Lon
JR16003	130	2	KRA	F	63	Frozen	Y	1	CT	RMT25	-54.576	-45.107
JR16003	130	2	KRA	M	63	Frozen	Y	1	CT	RMT25	-54.576	-45.107
JR16003	130	2	KRA	M	55	Frozen	Y	1	CT	RMT25	-54.576	-45.107
JR16003	171	1	KRA	F	55	Frozen	Y	1	CT	RMT25	-56.719	-56.858
JR16003	147	1	KRA	M	51	Frozen	Inc	NA	CT	RMT25	-53.951	-49.247
JR16003	171	1	KRA	M	46	Frozen	R	1	CT	RMT25	-56.719	-56.858
JR16003	130	2	KRA	J	36	Frozen	R	1	CT	RMT25	-54.576	-45.107
JR16003	171	1	KRA	U	38	Frozen	Inc	NA	CT	RMT25	-56.719	-56.858
JR16003	164	1	KRA	U	39	Frozen	R	1	CT	RMT25	-53.292	-52.200
JR16003	171	1	KRA	J	30	Frozen	Y	1	CT	RMT25	-56.719	-56.858
JR15004	60	2	ELN	F	99	Frozen	N	0	CT	RMT25	-59.997	-47.231
JR15004	60	2	ELN	M	96	Frozen	N	0	CT	RMT25	-59.997	-47.231
JR15004	60	2	ELN	F	76	Frozen	N	0	CT	RMT25	-59.997	-47.231
JR15004	60	2	ELN	F	77	Frozen	N	0	CT	RMT25	-59.997	-47.231
JR15004	60	2	ELN	M	68	Frozen	N	0	CT	RMT25	-59.997	-47.231
JR15004	91	1	ELN	F	64	Frozen	N	0	CT	RMT25	-60.257	-46.213
JR15004	96	2	ELN	F	61	Frozen	N	0	CT	RMT25	-60.340	-46.662
JR15004	91	1	ELN	F	60	Frozen	Y	1	CT	RMT25	-60.257	-46.213
JR15004	73	2	ELN	J	45	Frozen	Inc	NA	CT	RMT25	-60.119	-46.081
JR15004	72	2	ELN	J	45	Frozen	Y	1	CT	RMT25	-60.115	-46.078
JR177	165	2	ELN	U	45	IKI	Y	1	CT	RMT25	-59.684	-44.170
JR177	165	2	ELN	U	45	IKI	Y	1	CT	RMT25	-59.684	-44.170
JR177	165	2	ELN	U	51	IKI	N	0	CT	RMT25	-59.684	-44.170
JR177	165	2	ELN	U	47	IKI	Y	1	CT	RMT25	-59.684	-44.170
JR177	165	2	ELN	U	50	IKI	R	1	CT	RMT25	-59.684	-44.170
JR177	300	2	ELN	F	93	IKI	N	0	CT	RMT25	-52.879	-40.153
JR177	300	2	ELN	F	84	IKI	N	0	CT	RMT25	-52.879	-40.153
JR177	300	2	ELN	M	61	IKI	N	0	CT	RMT25	-52.879	-40.153
JR177	300	2	ELN	U	47	IKI	Inc	NA	CT	RMT25	-52.879	-40.153
JR177	300	2	ELN	F	59	IKI	N	0	CT	RMT25	-52.879	-40.153
JR177	300	2	ELN	F	70	IKI	N	0	CT	RMT25	-52.879	-40.153
JR177	379	1	ELN	M	73	IKI	N	0	CT	RMT25	-53.590	-37.659
JR177	379	1	ELN	M	67	IKI	D	NA	CT	RMT25	-53.590	-37.659
JR177	379	1	ELN	F	66	IKI	Inc	NA	CT	RMT25	-53.590	-37.659
JR177	379	1	ELN	F	44	IKI	Y	1	CT	RMT25	-53.590	-37.659
JR177	379	1	ELN	F	64	IKI	N	0	CT	RMT25	-53.590	-37.659
JR177	379	1	ELN	M	66	IKI	N	0	CT	RMT25	-53.590	-37.659
JR177	357	1	ELC	F	81	IKI	R	1	CT	RMT25	-50.520	-34.081
JR177	357	1	ELC	M	78	IKI	Y	1	CT	RMT25	-50.520	-34.081
JR177	357	1	ELC	F	76	IKI	R	1	CT	RMT25	-50.520	-34.081

JR177	357	1	ELC	M	76	IKI	R	1	CT	RMT25	-50.520	-34.081
JR177	357	1	ELC	F	82	IKI	R	1	CT	RMT25	-50.520	-34.081
JR177	357	1	ELC	F	71	IKI	R	1	CT	RMT25	-50.520	-34.081
JR177	357	1	ELC	M	74	IKI	Y	1	CT	RMT25	-50.520	-34.081
JR177	379	1	GYR		98	Frozen	N	0	X-ray	RMT25	-53.590	-37.659
JR177	379	1	GYR		110	Frozen	N	0	X-ray	RMT25	-53.590	-37.659
JR177	379	1	GYR		99	Frozen	N	0	X-ray	RMT25	-53.590	-37.659
JR177	379	1	GYR		77	Frozen	N	0	X-ray	RMT25	-53.590	-37.659
JR177	379	1	GYR		107	Frozen	N	0	X-ray	RMT25	-53.590	-37.659
JR15004	73	2	GYR		68	Frozen	N	0	X-ray	RMT25	-60.119	-46.081
JR15004	73	2	GYR		69	Frozen	N	0	X-ray	RMT25	-60.119	-46.081
JR15004	73	2	GYR		74	Frozen	N	0	X-ray	RMT25	-60.119	-46.081
JR15004	73	2	GYR		96	Frozen	N	0	X-ray	RMT25	-60.119	-46.081
JR15004	73	2	GYR		70	Frozen	N	0	X-ray	RMT25	-60.119	-46.081
JR15004	61	1	GYR		112	Frozen	N	0	X-ray	RMT25	-59.975	-47.202
JR15004	61	1	GYR		113	Frozen	N	0	X-ray	RMT25	-59.975	-47.202
JR15004	61	1	GYR		101	Frozen	N	0	X-ray	RMT25	-59.975	-47.202
JR15004	61	1	GYR		115	Frozen	N	0	X-ray	RMT25	-59.975	-47.202
JR15004	61	1	GYR		123	Frozen	N	0	X-ray	RMT25	-59.975	-47.202
JR177	305	2	GYF	F	37	Frozen	Inc	NA	X-ray	RMT25	-52.870	-40.085
JR177	305	2	GYF	F	55	Frozen	N	0	X-ray	RMT25	-52.870	-40.085
JR177	305	2	GYF	F	37	Frozen	D	NA	X-ray	RMT25	-52.870	-40.085
JR177	305	2	GYF	M	63	Frozen	N	0	X-ray	RMT25	-52.870	-40.085
JR177	305	2	GYF	F	40	Frozen	Inc	NA	X-ray	RMT25	-52.870	-40.085
JR16003	164	2	GYF		75	Frozen	N	0	X-ray	RMT25	-53.301	-52.207
JR16003	164	2	GYF	F	73	Frozen	N	0	X-ray	RMT25	-53.301	-52.207
JR16003	164	2	GYF		82	Frozen	N	0	X-ray	RMT25	-53.301	-52.207
JR177	378	1	GYN		146	Frozen	N	0	X-ray	RMT25	-53.587	-37.662
JR177	378	1	GYN		131	Frozen	N	0	CT	RMT25	-53.587	-37.662
JR177	378	1	GYN		133	Frozen	N	0	X-ray	RMT25	-53.587	-37.662
JR177	378	1	GYN		126	Frozen	N	0	X-ray	RMT25	-53.587	-37.662
JR177	378	1	GYN		141	Frozen	N	0	X-ray	RMT25	-53.587	-37.662
JR15004	104	1	PRM		41	IKI	Y	1	CT	RMT8	-57.027	-51.601
JR15004	104	1	PRM		37	IKI	Y	1	CT	RMT8	-57.027	-51.601
JR15004	104	1	PRM		43	IKI	Y	1	CT	RMT8	-57.027	-51.601
JR15004	104	1	PRM		42	IKI	Y	1	CT	RMT8	-57.027	-51.601
JR15004	104	1	PRM		43	IKI	Y	1	CT	RMT8	-57.027	-51.601
JR15004	104	1	PRM		42	IKI	R	1	CT	RMT8	-57.027	-51.601
JR15004	104	1	PRM		51	IKI	R	1	CT	RMT8	-57.027	-51.601
JR15004	104	1	PRM		43	IKI	R	1	CT	RMT8	-57.027	-51.601
JR15004	104	1	PRM		44	IKI	R	1	CT	RMT8	-57.027	-51.601
JR15004	104	1	PRM		42	IKI	R	1	CT	RMT8	-57.027	-51.601
JR15004	104	1	PRM		45	IKI	R	1	CT	RMT8	-57.027	-51.601
JR15004	60	2	ELN	F	79	Fresh	N	0	X-ray	RMT25	-59.997	-47.231
JR15004	60	2	ELN	M	77	Fresh	N	0	X-ray	RMT25	-59.997	-47.231
JR15004	60	2	ELN		70	Fresh	N	0	X-ray	RMT25	-59.997	-47.231
JR15004	60	2	ELN		103	Fresh	N	0	X-ray	RMT25	-59.997	-47.231
JR15004	60	2	ELN		92	Fresh	N	0	X-ray	RMT25	-59.997	-47.231
JR15004	60	2	GYR		81	Fresh	N	0	X-ray	RMT25	-59.997	-47.231
JR15004	60	2	GYR		108	Fresh	N	0	X-ray	RMT25	-59.997	-47.231
JR15004	60	2	GYR		115	Fresh	N	0	X-ray	RMT25	-59.997	-47.231
JR15004	60	2	GYR		104	Fresh	N	0	X-ray	RMT25	-59.997	-47.231
JR15004	60	2	GYR		80	Fresh	N	0	X-ray	RMT25	-59.997	-47.231

JR15004	65	1	ELN	M	66	Fresh	N	0	X-ray	RMT25	-60.014	-46.614
JR15004	65	1	ELN	F	88	Fresh	N	0	X-ray	RMT25	-60.014	-46.614
JR15004	65	1	ELN	F	88	Fresh	N	0	X-ray	RMT25	-60.014	-46.614
JR15004	65	1	ELN	F	82	Fresh	N	0	X-ray	RMT25	-60.014	-46.614
JR15004	65	1	ELN	F	66	Fresh	N	0	X-ray	RMT25	-60.014	-46.614
JR15004	65	1	ELN	F	78	Fresh	N	0	X-ray	RMT25	-60.014	-46.614
JR15004	65	1	ELN	F	81	Fresh	N	0	X-ray	RMT25	-60.014	-46.614
JR15004	65	1	ELN	F	88	Fresh	N	0	X-ray	RMT25	-60.014	-46.614
JR15004	65	1	ELN	F	71	Fresh	N	0	X-ray	RMT25	-60.014	-46.614
JR15004	65	1	ELN	F	66	Fresh	N	0	X-ray	RMT25	-60.014	-46.614
JR15004	72	2	ELN	J	49	Fresh	Y	1	X-ray	RMT25	-60.115	-46.078
JR15004	72	2	ELN	M	63	Fresh	N	0	X-ray	RMT25	-60.115	-46.078
JR15004	72	2	ELN	F	69	Fresh	N	0	X-ray	RMT25	-60.115	-46.078
JR15004	72	2	ELN	M	62	Fresh	N	0	X-ray	RMT25	-60.115	-46.078
JR15004	73	2	PRM	M	40	Fresh	R	1	X-ray	RMT25	-60.115	-46.078
JR15004	73	2	ELN	M	70	Fresh	N	0	X-ray	RMT25	-60.115	-46.078
JR15004	91	1	ELN	F	59	Fresh	N	0	X-ray	RMT25	-60.257	-46.213
JR15004	91	1	ELN	F	66	Fresh	N	0	X-ray	RMT25	-60.257	-46.213
JR15004	91	1	ELN	F	64	Fresh	N	0	X-ray	RMT25	-60.257	-46.213
JR15004	96	2	GYN		139	Fresh	N	0	X-ray	RMT25	-60.340	-46.662
JR15004	96	2	GYN		141	Fresh	N	0	X-ray	RMT25	-60.340	-46.662
JR15004	96	2	GYN		135	Fresh	N	0	X-ray	RMT25	-60.340	-46.662
JR15004	96	2	GYN		153	Fresh	N	0	X-ray	RMT25	-60.340	-46.662
JR15004	96	2	GYN		150	Fresh	N	0	X-ray	RMT25	-60.340	-46.662
JR15004	96	2	GYN		144	Fresh	N	0	X-ray	RMT25	-60.340	-46.662
JR15004	96	2	GYN		124	Fresh	N	0	X-ray	RMT25	-60.340	-46.662
JR15004	96	2	GYN		141	Fresh	N	0	X-ray	RMT25	-60.340	-46.662
JR15004	96	2	GYN		129	Fresh	N	0	X-ray	RMT25	-60.340	-46.662
JR15004	96	2	ELN	F	60	Fresh	N	0	X-ray	RMT25	-60.340	-46.662
JR15004	96	2	ELN	M	65	Fresh	N	0	X-ray	RMT25	-60.340	-46.662
JR15004	104	1	ELC	F	74	Fresh	R	1	X-ray	RMT8	-57.027	-51.601
JR15004	104	1	ELC	F	77	Fresh	R	1	X-ray	RMT8	-57.027	-51.601
JR15004	105	2	ELC		83	Fresh	R	1	X-ray	RMT8	-57.026	-51.553
JR15004	105	2	ELC		86	Fresh	R	1	X-ray	RMT8	-57.026	-51.553
JR15004	105	2	ELC		85	Fresh	R	1	X-ray	RMT8	-57.026	-51.553
JR15004	105	1	ELC	F	75	Fresh	R	1	X-ray	RMT8	-57.041	-51.519
JR15004	105	1	ELC		73	Fresh	R	1	X-ray	RMT8	-57.041	-51.519
JR15004	105	1	PRM	M	43	Fresh	Y	1	X-ray	RMT8	-57.041	-51.519
JR15004	105	1	PRM	M	47	Fresh	Y	1	X-ray	RMT8	-57.041	-51.519
JR15004	105	1	PRM	F	48	Fresh	R	1	X-ray	RMT8	-57.041	-51.519
JR15004	106	2	ELC		76	Fresh	R	1	X-ray	RMT8	-57.024	-51.545
JR15004	106	2	ELC		84	Fresh	R	1	X-ray	RMT8	-57.024	-51.545
JR15004	106	2	ELC		77	Fresh	R	1	X-ray	RMT8	-57.024	-51.545
JR15004	106	2	ELC		76	Fresh	R	1	X-ray	RMT8	-57.024	-51.545
JR15004	106	2	ELC		86	Fresh	R	1	X-ray	RMT8	-57.024	-51.545
JR15004	106	2	ELC		70	Fresh	R	1	X-ray	RMT8	-57.024	-51.545
JR15004	106	2	ELC		80	Fresh	R	1	X-ray	RMT8	-57.024	-51.545
JR15004	106	2	ELN	J	43	Fresh	N	0	X-ray	RMT8	-57.024	-51.545
JR15004	106	2	ELN	J	32	Fresh	Y	1	X-ray	RMT8	-57.024	-51.545
JR15004	106	2	PRM		51	Fresh	Y	1	X-ray	RMT8	-57.024	-51.545
JR16003	39	2	ELN	F	44	Fresh	Y	1	Dis	RMT8	-53.540	-39.250
JR16003	39	2	PRM	F	51	Fresh	Y	1	Dis	RMT8	-53.540	-39.250
JR16003	89	1	PRM		29	Fresh	Y	1	Dis	MOC	-52.840	-40.211

JR16003	129	2	ELC		79	Fresh	Y	1	Dis	RMT25	-54.653	-45.188
JR16003	129	2	ELC		76	Fresh	Y	1	Dis	RMT25	-54.653	-45.188
JR16003	129	2	ELC		79	Fresh	Y	1	Dis	RMT25	-54.653	-45.188
JR16003	129	2	ELC		80	Fresh	Y	1	Dis	RMT25	-54.653	-45.188
JR16003	129	2	ELC		72	Fresh	Y	1	Dis	RMT25	-54.653	-45.188
JR16003	129	2	ELC		78	Fresh	Y	1	Dis	RMT25	-54.653	-45.188
JR16003	129	2	ELC		77	Fresh	Y	1	Dis	RMT25	-54.653	-45.188
JR16003	129	2	ELN		65	Fresh	Y	1	Dis	RMT25	-54.653	-45.188
JR16003	129	2	ELN		95	Fresh	N	0	Dis	RMT25	-54.653	-45.188
JR16003	129	2	PRM	M	62	Fresh	Y	1	Dis	RMT25	-54.653	-45.188
JR16003	129	2	PRM	M	61	Fresh	Y	1	Dis	RMT25	-54.653	-45.188
JR16003	129	2	PRM	F	53	Fresh	Y	1	Dis	RMT25	-54.653	-45.188
JR16003	129	2	PRM	F	60	Fresh	Y	1	Dis	RMT25	-54.653	-45.188
JR16003	129	2	PRM	F	57	Fresh	Y	1	Dis	RMT25	-54.653	-45.188
JR16003	143	2	KRA		42	Fresh	Y	1	Dis	MOC	-53.930	-49.164
JR16003	143	1	ELN		27	Fresh	Y	1	Dis	MOC	-53.932	-49.112
JR16003	146	2	KRA	F	51	Fresh	Y	1	Dis	RMT25	-53.958	-49.197
JR16003	146	2	KRA	M	45	Fresh	Y	1	Dis	RMT25	-53.958	-49.197
JR16003	146	2	KRA	M	40	Fresh	Y	1	Dis	RMT25	-53.958	-49.197
JR16003	146	2	KRA	M	45	Fresh	Y	1	Dis	RMT25	-53.958	-49.197
JR16003	146	2	KRA	M	52	Fresh	Y	1	Dis	RMT25	-53.958	-49.197
JR16003	146	2	GYR		105	Fresh	N	0	Dis	RMT25	-53.958	-49.197
JR16003	146	1	KRA	F	68	Fresh	Y	1	Dis	RMT25	-53.948	-49.180
JR16003	146	1	KRA	F	62	Fresh	Y	1	Dis	RMT25	-53.948	-49.180
JR16003	146	1	KRA	M	64	Fresh	Y	1	Dis	RMT25	-53.948	-49.180
JR16003	147	1	KRA	F	48	Fresh	Y	1	Dis	RMT25	-53.951	-49.247
JR16003	147	1	KRA	F	48	Fresh	Y	1	Dis	RMT25	-53.951	-49.247
JR16003	147	1	KRA	F	49	Fresh	N	0	Dis	RMT25	-53.951	-49.247
JR16003	147	1	KRA	F	48	Fresh	Y	1	Dis	RMT25	-53.951	-49.247
JR16003	147	2	PRM	F	59	Fresh	Y	1	Dis	RMT25	-53.929	-49.262
JR16003	147	2	PRM	F	56	Fresh	Y	1	Dis	RMT25	-53.929	-49.262
JR16003	147	2	PRM	M	57	Fresh	Y	1	Dis	RMT25	-53.929	-49.262
JR16003	147	2	PRM	F	60	Fresh	Y	1	Dis	RMT25	-53.929	-49.262
JR16003	164	2	GYF		76	Fresh	N	0	Dis	RMT25	-53.301	-52.207
JR16003	164	2	PRM	M	54	Fresh	Y	1	Dis	RMT25	-53.301	-52.207
JR16003	164	2	PRM	M	51	Fresh	Y	1	Dis	RMT25	-53.301	-52.207
JR16003	164	2	PRM	J	32	Fresh	Y	1	Dis	RMT25	-53.301	-52.207
JR16003	164	2	PRM	M	37	Fresh	Y	1	Dis	RMT25	-53.301	-52.207
JR16003	164	2	PRM	F	50	Fresh	Y	1	Dis	RMT25	-53.301	-52.207
JR16003	164	2	KRA	M	48	Fresh	N	0	Dis	RMT25	-53.301	-52.207
JR16003	171	1	ELN	J	42	Fresh	Y	1	Dis	RMT25	-56.719	-56.858
JR16003	171	1	KRA	M	65	Fresh	Y	1	Dis	RMT25	-56.719	-56.858
JR16003	171	1	KRA	M	58	Fresh	Y	1	Dis	RMT25	-56.719	-56.858
JR16003	171	1	KRA	F	64	Fresh	Y	1	Dis	RMT25	-56.719	-56.858
JR16003	171	1	KRA	M	67	Fresh	Y	1	Dis	RMT25	-56.719	-56.858
JR16003	171	1	ELN	J	41	Fresh	N	0	Dis	RMT25	-56.719	-56.858
JR16003	171	1	KRA	f	37	Frozen	N	0	CT	RMT25	-56.719	-56.858
JR16003	171	1	KRA	j	33	Frozen	R	1	CT	RMT25	-56.719	-56.858
JR16003	171	2	KRA	m	31	Frozen	Y	1	CT	RMT25	-56.731	-56.866
JR16003	130	1	KRA	j	39	Frozen	Inc	NA	CT	RMT25	-54.594	-45.118
JR16003	171	1	KRA	f	38	Frozen	Y	1	CT	RMT25	-56.719	-56.858
JR16003	171	1	KRA	f	47	Frozen	R	1	CT	RMT25	-56.719	-56.858
JR16003	171	1	KRA	m	49	Frozen	N	0	CT	RMT25	-56.719	-56.858

JR16003	171	1	KRA	f	43	Frozen	Y	1	CT	RMT25	-56.719	-56.858
JR16003	147	1	KRA	f	45	Frozen	Inc	NA	CT	RMT25	-53.951	-49.247
JR16003	171	1	KRA	m	50	Frozen	Inc	NA	X-ray	RMT25	-56.719	-56.858
JR16003	164	2	KRA	f	52	Frozen	D	NA	CT	RMT25	-53.301	-52.207
JR16003	171	1	KRA	f	53	Frozen	N	0	X-ray	RMT25	-56.719	-56.858
JR16003	163	2	KRA	f	54	Frozen	N	0	CT	RMT25	-53.267	-52.174
JR16003	163	2	KRA	f	57	Frozen	R	1	X-ray	RMT25	-53.267	-52.174
JR16003	171	1	KRA	m	55	Frozen	D	NA	X-ray	RMT25	-56.719	-56.858
JR16003	130	2	KRA	f	70	Frozen	R	1	X-ray	RMT25	-54.576	-45.107
JR16003	130	2	KRA	f	70	Frozen	R	1	CT	RMT25	-54.576	-45.107
JR16003	130	2	KRA	f	62	Frozen	Y	1	CT	RMT25	-54.576	-45.107
JR16003	130	2	KRA	m	71	Frozen	Inc	NA	X-ray	RMT25	-54.576	-45.107
JR16003	130	2	GYF	m	70	Frozen	N	0	CT	RMT25	-54.576	-45.107
JR16003	147	2	GYF	m	69	Frozen	N	0	CT	RMT25	-53.929	-49.262
JR16003	147	2	GYF	m	70	Frozen	N	0	CT	RMT25	-53.929	-49.262
JR16003	147	2	GYF		74	Frozen	N	0	CT	RMT25	-53.929	-49.262
JR16003	147	2	GYF		84	Frozen	N	0	CT	RMT25	-53.929	-49.262
JR200	115	1	GYF		79	Frozen	D	NA	CT	RMT25	-56.803	-42.247
JR200	141	1	GYF		80	Frozen	N	0	CT	RMT25	-55.256	-41.356
JR200	185	2	GYF		67	Frozen	D	NA	CT	RMT25	-52.826	-39.877

Table S2. Decision tree for classifying presence absence of gas in species, based on condition. All scans deemed damaged or inconclusive were excluded from further analysis. Images here are representative 2D slices of CT scans. All CT were checked for gas as 3D reconstructions. Other data used to ascertain presence/absence of gas were soft tissue x-ray and dissection.

**Fish condition**	**Gas bearing bladder**	**Gas binary**	**Example image**
Gas contained in un-ruptured bladder.<br>All other tissue good.	Yes	1	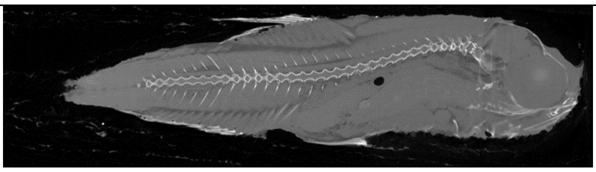<br>ELN_030
Gas in swimbladder region and /or abdomen due to apparent rupturing.<br>Surrounding tissue good.	Yes (ruptured)	1	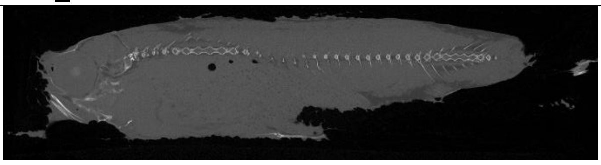<br>KRA_315<br>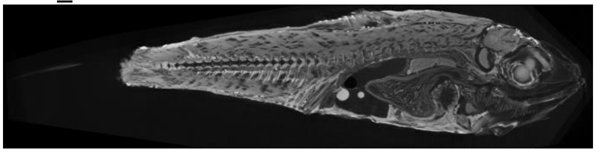<br>PRM_009<br>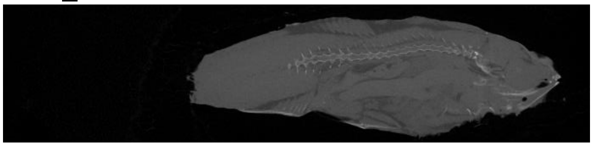<br>KRA_719
No gas in swimbladder.<br>No gas in abdomen.<br>All surrounding tissue good.	No	0	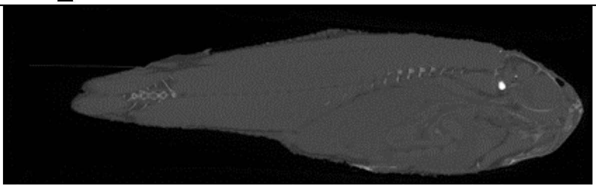<br>ELN_059
No gas visible in swimbladder region.<br>Gas entrainment along skeletal tissues to upper body and extremities (muscle tissue striation).	No	0	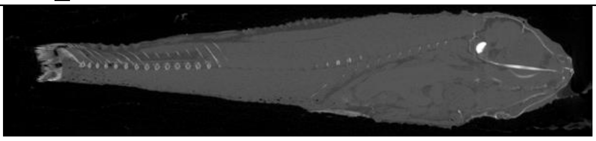<br>ELN_060
No gas visible in swimbladder region.<br>Gas entrainment via orifices.	No	0	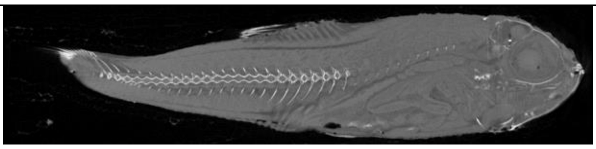<br>ELN_063<br>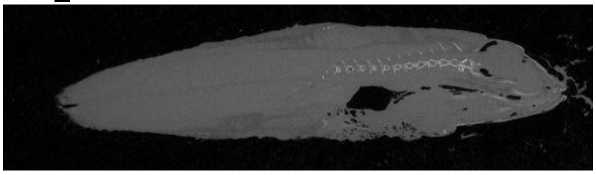<br>GYF_Cam049

Gas entrainment throughout fish tissues OR damage to abdomen likely to have resulted in gas entrainment.	Damaged	NA	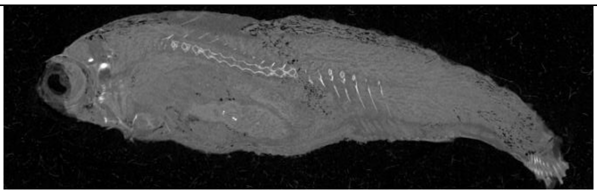 KRA_Cam711
Scan inconclusive / lack of tissue or cavity definition	Inconclusive	NA	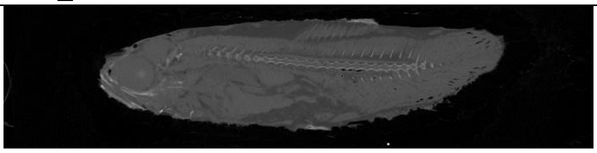 KRA_569

Table S3. Summary of swimbladder condition of myctophid species and data source. Species: ELN – *Electrona antarctica*, ELC – *E. carlsbergi*, GYR – Gymnoscopelus braueri, GYF – *G. fraseri*, GYN – *G. nicholsi*, KRA – *Krefftichthys anderssoni*, PRM – *Protomyctophum bolini*. Gas (+), Non-gas (-), Damaged sample excluded from analysis (D), inconclusive scan excluded from analysis (Inc.).

**Species**	**CT**				**X-ray**				**Dissection**				**Total**		
	+	-	D	Inc.	+	-	D	Inc.	+	-	D	Inc.	**+**	**-**	**Excluded**
**ELN**	7	16	1	3	2	25			4	2			**13**	**43**	**4**
**ELC**	7				14				7				**28**	**0**	**0**
**GYR**						20				1			**0**	**21**	**0**
**GYF**		6	2			5	1	2		1			**0**	**12**	**5**
**GYN**		1				13							**0**	**14**	**0**
**KRA**	15	3	1	4	2	1	1	2	16	2			**33**	**6**	**8**
**PRM**	11				5				16				**32**	**0**	**0**
**Total**	40	26	4	7	23	64	2	4	43	6	0	0	**106**	**96**	**17**

Table S4. Species used in community assessment and gas bladder condition applied. The most abundant mesopelagic fish species were identified for latitudinal community analysis based on Scotia Sea RMT25 nets data from cruises JR161, JR177, JR200, JR15004 and JR16003. The percentage community contribution (%) is based on the percentage of individuals sampled from all available data, where n is the total number of individuals. Species included account for 94.6% (n = 10674) of individuals, the remaining 5.4% of individuals (n = 609) was comprised of 41 species of mesopelagic fish. Of the potential *Cyclothone* species in the Scotia Sea, the fat-invested *Cyclothone microdon* is most prevalent in the region (Ainley et al. 1986; Donnelly et al. 1990]. Since *Cyclothone* species were not identified to species, we base our analyses on an assumption that the dominant species in catches is *C. microdon* and therefore *Cyclothone* individuals in the RMT25 catches are predominantly fat-invested.

Species	Family	Swimbladder	Source	n	%	
Electrona antarctica	Myctophidae	Gas to None	Current study	2728	24.18	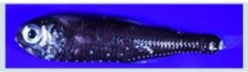
Electrona carlsbergi		Gas	Current study	704	6.24	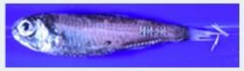
Gymnoscopelus braueri		Regressed	Current study	1890	16.75	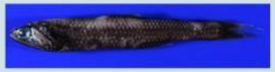
Gymnoscopelus fraseri		Regressed	Current study	187	1.66	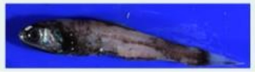
Gymnoscopelus nicholsi		Regressed	Current study	118	1.05	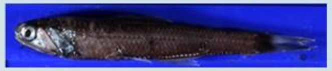
Protomyctophum bolini		Gas	Current study	876	7.76	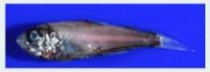
Protomyctophum tenisoni		Gas	Marshall (1960)	324	2.87	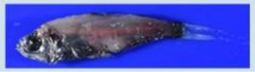
Krefftichthys anderssoni		Gas	Current study	1307	11.58	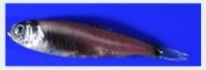
Bathylagus spp.	Bathylagidae	No swimbladder	Marshall (1960)	1626	14.41	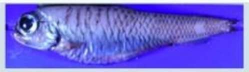
Cyclothone spp.	Gonostomatidae	Fat invested	Marshall (1960)	711	6.30	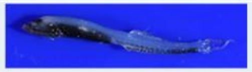
Notolepis spp.	Paralepididae	No swimbladder	Froese & Pauly. eds. (2018)	203	1.80	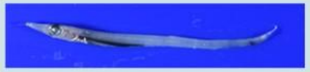

Ainley DG, Fraser WR, Sullivan CW, Torres JJ, Hopkins TL, Smith WO. 1986 Antarctic mesopelagic micronekton: Evidence from seabirds that pack ice affects community structure. *Science* **232**, 847-849. (doi:10.1126/science.232.4752.847).

Donnelly J, Torres JJ, Hopkins TL, Lancraft TM. 1990 Proximate composition of Antarctic mesopelagic fishes. *Marine Biology* **106**, 13-23. (doi:10.1007/bf02114670).

Froese, R. and Pauly D. 2018 FishBase. World Wide Web electronic publication. www.fishbase.org.

Marshall, NB. 1960 Swimbladder structure of deep-sea fishes in relation to their systematics and biology. *Discovery Reports* **31**, 1-122.

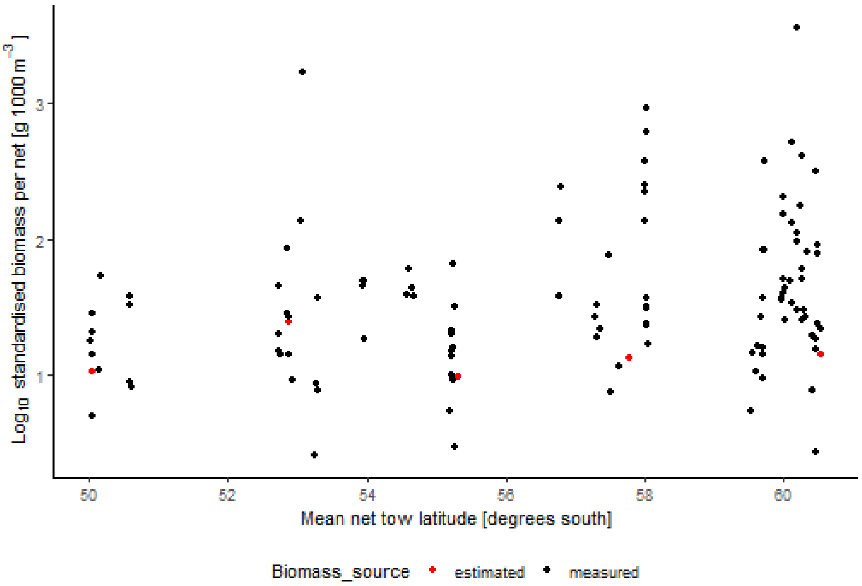

Figure S2. Log_{10} total net biomass of all fauna (both fish and invertebrate) captured in the night RMT25 nets used in the current study, plotted against mean net tow latitude. All nets were standardised for tow speed and duration.

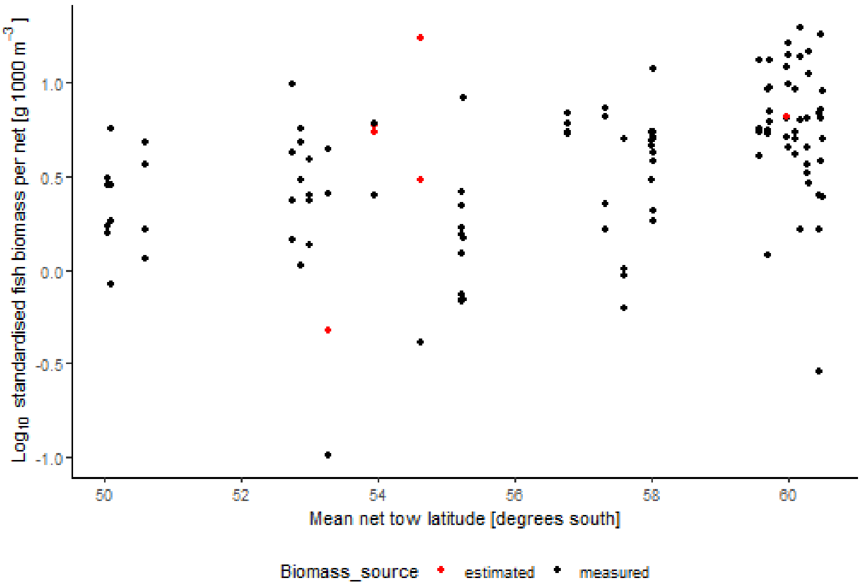

Figure S3. Log_{10} total fish biomass captured in the night RMT25 nets used in the current study, plotted against mean net tow latitude. Fish species included in fish biomass assessment are those listed in Table S4 only. All nets were standardised for tow speed and duration.

Where catch biomass weights were missing, a mean weight for an individual from each species was calculated from combined JR161, JR177 and JR200 RMT25 catch biomass data. Abundances were then multiplied by mean weight to estimate biomass. Net samples containing some estimated values are coloured red. Ships scale precision is to the nearest gram. Where species weights were recorded as < 1 g these were set to 0.5 g for the purposes of calculation.

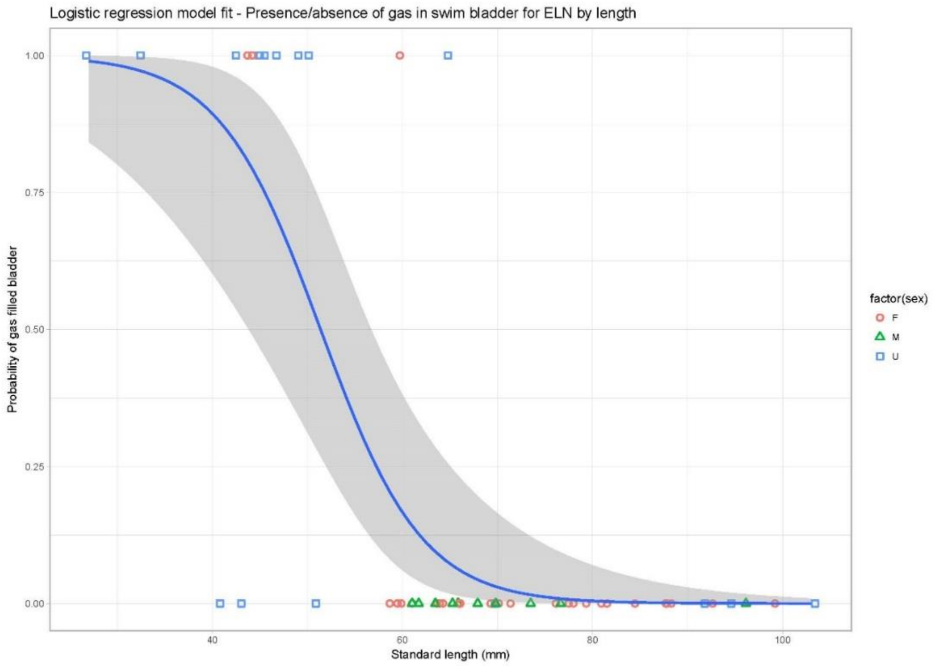

Figure S4. Logistic regression model fit predicting the probability of presence of gas in *Electrona antarctica* swim bladders, by standard length (mm). Shading indicates 95% confidence intervals. Jitter added to data points (width 0.5) for visualisation. Sex indicated by points, red circle: female, green triangle: male, blue square: unclassified.

Standard length was a highly significant predictor of the presence of gas, p-value <0.0001, null deviance 60.687 on 55 df, residual deviance 27.866 on 54 df. Using values of intercept (a) and slope (b) defined by the model, the estimated standard length of *E. antarctica*, where probability (p) of presence of gas was 0.5 (SL0.5) was calculated as follows:

$$SL_{0.5} = \frac{log\left(\frac{p}{1-p}\right) - a}{b} = \frac{log\left(\frac{0.5}{1-0.5}\right) - 9.614876}{-0.18714} = 51.378\,mm$$

Prediction of standard length with gas presence probability of 0.5 was 51.378 mm, which was subsequently used to assess the proportion of the *E. antarctica* community likely to be gas bearing.

Binomial glm coefficients based on logit link function

**Model**	**term**	**estimate**	**std.error**	**statistic**	**p.value**	**link**
**1**	(Intercept)	9.614876	2.813797	3.417047	0.000633	logit
**1**	standard_length_mm	-0.18714	0.051166	-3.65747	0.000255	logit

Binomial glm outputs based on logit link function

**Model**	**null.deviance**	**df.null**	**logLik**	**AIC**	**BIC**	**deviance**	**df.residual**	**link**
**1**	60.6875	55	-13.9332	31.86647	35.91717	27.86647	54	logit
